# Evaluation of FAU-type Zeolite Membrane Stability in Transesterification Reaction Conditions

**DOI:** 10.3390/membranes13010068

**Published:** 2023-01-05

**Authors:** Ayumi Ikeda, Wakako Matsuura, Chie Abe, Sean-Thomas Bourne Lundin, Yasuhisa Hasegawa

**Affiliations:** National Institute of Advanced Industrial Science and Technology (AIST), Research Institute of Chemical Process Technology, 4-2-1 Nigatake, Miyagino-ku, Sendai 983-8551, Japan

**Keywords:** methanol permselective membrane, vapor exposure test, organic vapor stability

## Abstract

The transesterification conversion of methyl ether can be enhanced by the removal of the byproduct methanol using methanol permselective faujasite (FAU-type) zeolite membranes. However, the authors previously observed that the methanol flux during the transesterification reaction was lower than the predicted flux. Therefore, this study investigated the stability of FAU-type zeolite membranes in the presence of organic components associated with the transesterification reaction of methyl hexanoate and 1-hexanol. The stability was defined in terms of changes in methanol permeance and zeolite structure. The effect of reaction components (methanol, 1-hexanol, methyl hexanoate, and hexyl hexanoate) on the FAU-type zeolite structure and the methanol permeation performance of the FAU-type zeolite membranes were evaluated to find the component causing the lower methanol flux. From these results, two esters were found to adsorb strongly on the FAU-type zeolite. The methanol flux of the FAU-type zeolite membrane was examined after vapor exposure of each of the four reaction chemicals at 373 K for 8 h. In the case of methyl hexanoate and hexyl hexanoate vapor exposure, the methanol flux was reduced by about 75% compared to the initial flux of 15 kg m^−2^ h^−1^. These results indicated methanol permeation performance was inhibited by the adsorption of esters.

## 1. Introduction

Methanol is an important chemical substance as fuel, solvent, raw materials for fine chemical. Separation of methanol from organic mixtures in chemical process is required. Methanol forms azeotropic mixtures with numerous solvents and esters [1]. Therefore, membrane separation is suitable for the separation technique for methanol separation.

Methanol permselective membranes have been developed using materials such as polymer, zeolite, and silica [2,3,4,5,6]. These membranes have been reported methanol separation from organic solutions such as n-butyl acetate [2], methyl methacrylate [3], toluene [4] dimethyl carbonate [5], and methyl tert-butyl ether [5,6]. The authors have previously developed faujasite (FAU-type) zeolite membrane to separate methanol from alcohols and esters in transesterification reactions [7]. The developed FAU-type zeolite membrane with large pores displayed a high methanol flux of 10 kg m^−2^ h^−1^ and a separation factor of 6020 for a 10 wt% methanol/methyl hexanoate mixture. Moreover, the FAU-type zeolite membrane showed the high methanol flux and high methanol selectivity of C2-C6 alcohols and esters.

Over the last decade, transesterification reaction with methanol permselective membrane was reported and the conversion of methyl ether increased by shifting chemical equilibrium with methanol removal [8,9,10,11,12]. In the transesterification reaction where the reaction substrate is methyl ester, the methyl ester reacts with alcohol, causing the main chains to swap to form an ester with a large molecular weight and methanol. Selective removal of methanol from the reaction system increases the conversion. In several transesterification reactions, conversion was shown to be increased by methanol removal using permselective FAU-type zeolite membranes in the authors’ previous reports [8,9,10]. In the transesterification of methyl hexanoate and 1-hexanol at the initial molar ratio = 1, the conversion was increased from 57% to 78% at 373 K [10]. However, the methanol flux was lower than the predicted methanol flux by the separation performance of the quaternary vapor permeation test with simulant reaction solutions. Kumakiri et al. reported a lower methanol flux in the transesterification using FAU-type zeolite membranes compared with methanol/organic solution separation tests [13]. Therefore, it is important to investigate the causes of the declining methanol permeation of the FAU-type zeolite membrane in the transesterification reaction. Understanding the causes will enable to the development of high methanol permeation membranes suitable for the membrane-assisted transesterification reaction.

In this study, FAU-type zeolite membrane stability was evaluated under transesterification reaction conditions to understand what caused the previously reported decrease in the methanol flux. Additionally, changes in the zeolite structure of the FAU-type zeolite membrane were investigated. The transesterification reaction in which methyl hexanoate reacts with 1-hexanol to produce hexyl hexanoate and methanol was a model reaction. To investigate the adsorption of reaction components on the FAU-zeolite, the FAU-zeolite powder was immersed in methanol, 1-hexanol, methyl hexanoate, and hexyl hexanoate, respectively. Then, the immersed powders were measured by the TG-DTA analyzer. Effects of organic components vapor on the FAU-type zeolite membrane were evaluated by methanol permeation performance before and after vapor exposure tests. The membrane properties exposed to organic vapor were observed with SEM and XRD.

## 2. Materials and Methods

### 2.1. Materials

Sodium aluminate, sodium hydroxide, sodium silicate solution, and FAU (NaY)-type zeolite particles (HSZ-320NAA, TOSOH) were used to synthesize the zeolite membrane. The FAU-type zeolite particles had a Si/Al ratio of 2.8 and did not include any templating agents. Methanol, 2-propanol, 1-hexanol, methyl hexanoate, hexyl hexanoate, hexyl methyl ether, and hexyl ether were used in the pervaporation experiments and the vapor exposure tests. All reagents were purchased from FUJIFILM Wako (Tokyo, Japan) and used without further purification. A cation exchange resin (DOWEX 50Wx2 200–400 mesh, FUJIFILM Wako, Tokyo, Japan) was used as the catalyst to investigate the effect of an acid catalyst on membrane separation performance. The resin was washed three times with 2-propanol and vacuum-dried overnight in a nitrogen atmosphere.

### 2.2. Membrane Preparation

FAU-type zeolite membranes were synthesized on the outside of porous α-alumina support tubes (outer diameter: 3 mm, mean pore diameter: 0.3 μm, porosity: 50%) by a secondary growth method as previously reported [7,9]. A synthesis solution was prepared by stirring a mixture of sodium hydroxide, sodium aluminate, sodium silicate solution, and de-ionized water for 4 h at room temperature. The molar ratios of the synthesis solutions were 5 SiO_2_:1 Al_2_O_3_:7.5 Na_2_O:375 H_2_O and 5 SiO_2_:1 Al_2_O_3_:17 Na_2_O:1000 H_2_O. It did not matter which solution is used synthesize the membrane, as the Si/Al ratio of 1.3 and membrane thickness will be equivalent. FAU-type zeolite particles were rubbed on the outside of the support tube. Then, the tube was added to an autoclave filled with 30 g of the synthesis solution. The autoclave was placed horizontally in an oven at 363 K for 18 h or 16 h to grow the FAU-type zeolite crystals on the support tube. After cooling the autoclave, the tube was then washed with deionized water several times and dried overnight in open atmosphere at room temperature to obtain the FAU-type zeolite membrane.

The membrane morphology was observed using scanning electron microscopy (SEM, JEOL, JCM-6000, Tokyo Japan), and the composition was analyzed using an energy dispersive X-ray spectroscopy analyzer (EDX, JEOL, ED-2300, Tokyo, Japan) attached with the SEM. The crystal structure was identified by X-ray diffraction (XRD, Rigaku, Smart-Lab, Tokyo, Japan).

### 2.3. Thermal Analysis of Zeolite Powder after Immersion Treatment with Alcohols and Esters

Sample preparation for the immersion treatment involved immersion of 0.1 g of the FAU-type zeolite particles (hereafter referred to as FAU powders) into 10 g of methanol, 1-hexanol, methyl hexanoate, or hexyl hexanoate. Each solution was heated at 353 K for 1 h while stirring at 600 rpm, then the immersed FAU powders were dried at 353 K for 1 h. Thermal analysis of the FAU powders was measured by a thermogravimetry-differential thermal analyzer (TG-DTA, Rigaku, TG8120, Tokyo, Japan). The heating rate was 5 K min^−1^ and the feed gas was 100 mL min^−1^ of air.

### 2.4. Pervaporation Experiment

One end of the membrane was connected to a stainless-steel tube using a resin (GL Science, Torr seal, Tokyo, Japan), and the other end was capped. The effective membrane area for each test was 1.0 cm^2^. The test solutions were methanol or 10 wt% water/2-propanol mixture. For single methanol pervaporation tests, 150 g of methanol was added to a separable flask and heated at 333 K with continuous stirring at 600 rpm. For the pervaporation with 10 wt% water/2-propanol mixture, the test solution (1000 g) was added to a separable flask and heated at 348 K with continuous stirring at 1500 rpm. The membrane tube was immersed in the test solution and the inner side of the membrane tube was evacuated using a rotary pump to below 1 kPa. Helium was fed to the permeate side of the membrane at 3.0 mL min^−1^ as a standard. The gas composition in the evacuated stream was analyzed using mass spectrometry (Pfeiffer Vacuum, QME220, Asslar, Germany). The permeation flux, *J_i_*, of component *i* was calculated as follows [14]:

(1) $$J_{i}=\frac{N_{\mathrm{He}}}{S}\frac{y_{i}}{y_{He}},$$

where *N*_He_, *S*, and *y_i_* are the molar flow rate of helium, the membrane area, and the mole fraction of component *i* in the evacuated stream, respectively. The permeance of component *i*, *Q_i_*, was calculated using the following equation:

(2) $$Q_{i}=\frac{J_{i}}{p_{f,i}-p_{p,i}},$$

where *p*_f,*i*_ and *p*_p,*i*_ represent the partial vapor pressure of component *i* in the feed solution and the permeate side, respectively. The partial vapor pressure of component *i* was calculated using the Antoine constants and Wilson parameters listed in Table 1 [15]. The partial vapor pressure of component *i* is described as:

(3) $$p_{i}=x_{i}\gamma_{i}P_{i}{}^{\circ}=z_{i}P_{t},$$

where *x_i_* is the mole fraction of component *i* in the feed solution, *γ_i_* is the activity coefficient of component *i*, *P_i_*°is the vapor pressure of component *i*, *z_i_* is the mole component *i* in the vapor phase, and *P_t_* is the total vapor pressure. The total vapor pressure is calculated as follows:

(4) $$P_{t}=x_{i}\gamma_{i}P_{i}{}^{\circ}+x_{j}\gamma_{j}P_{j}{}^{\circ}.$$

The separation factor of water for 2-propanol, α, is defined as follows:

(5) $$\alpha_{\left(W/I\right)}=\frac{y_{W}/y_{I}}{x_{W}/x_{I}},$$

where *y_i_* is the mole fraction of component *i* in the permeate side, and the subscripts W and I indicate water and 2-propanol, respectively.

### 2.5. Vapor Exposure Test

The vapor exposure tests used the same apparatus for the transesterification reaction as the previous report [10]. 40 g of a single-component test solution (methanol, 1-hexanol, methyl hexanoate, hexyl hexanoate, hexyl methyl ether, or hexyl ether) was added into the separable flask and stirred at 600 rpm at 373 K for 8 h. The membrane was placed 1 cm above the liquid surface and the inside of the membrane was evacuated using a rotary pump. The test conditions were chosen to be consistent with the transesterification reaction.

## 3. Results

### 3.1. Characterization

**Figure 1:**
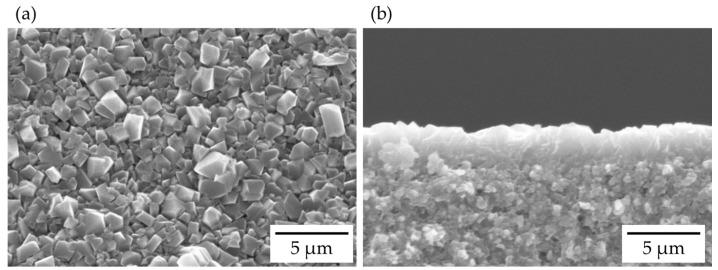
SEM images of (**a**) surface and (**b**) cross-section of FAU-type zeolite membranes (5 SiO_2_:1 Al_2_O_3_:7.5 Na_2_O:375 H_2_O, 18 h synthesis).

**Figure 2:**
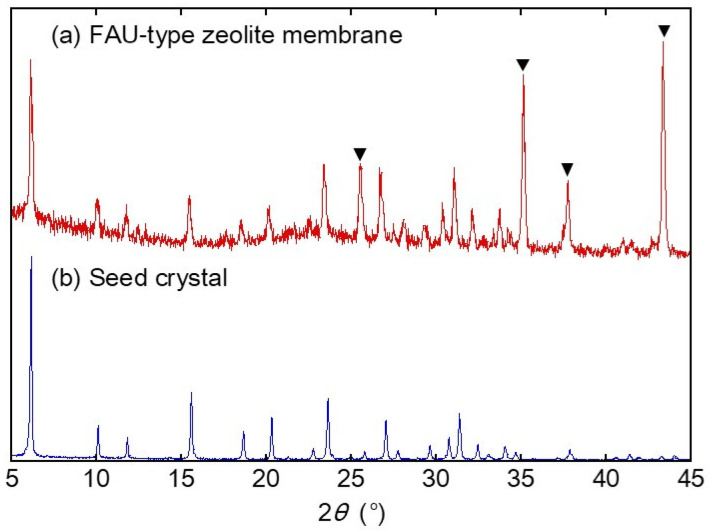
XRD patterns of (**a**) FAU-type zeolite membrane and (**b**) seed crystals (HSZ-320NAA). The triangle symbol (▼) represents XRD peaks of the α-alumina support.

Figure 1 shows the SEM images of the FAU-type zeolite membrane. The α-alumina support tube was completely covered with the polycrystalline layer, which had a grain size of 1–2 μm and a thickness of approximately 2.5 μm. As shown in Figure 2, peaks for both α-alumina and FAU-type zeolite were observed, which confirms the polycrystalline layer on the α-alumina support tube was FAU-type zeolite. The Si/Al ratio of the polycrystalline layer was determined to be 1.33 by EDX. The FAU-type zeolite membrane with the Si/Al ratio of 1.3 was successfully synthesized on the support tube.

### 3.2. TG-DTA Measurement of Zeolite Powder Immersed Alcohols and Esters

**Figure 3:**
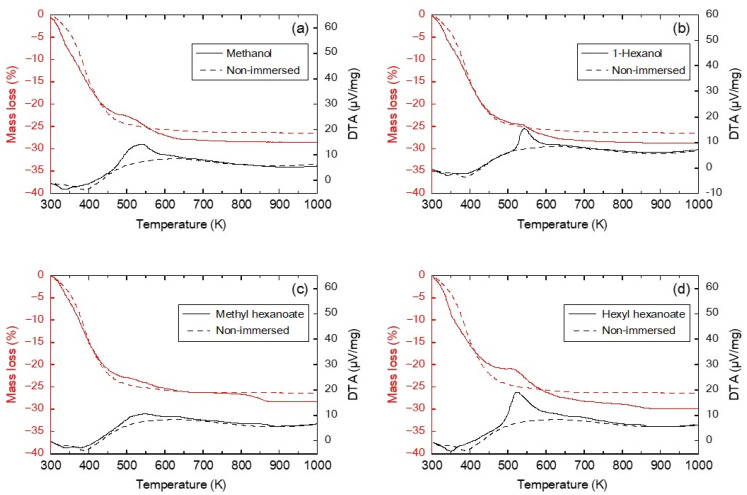
Thermal analysis of the FAU powder after immersion treatments in (**a**) methanol, (**b**) 1-hexanol, (**c**) methyl hexanoate, and (**d**) hexyl hexanoate. Non-immersed lines represent as a reference (dotted curves). The temperature ramp rate was 5 K min^−1^ and the feed was 100 mL min^−1^ air.

The mass change of the FAU powder and the effect of alcohols and esters used in the transesterification reaction on the FAU-type zeolite were evaluated by a TG-DTA analyzer. The trend of adsorption behavior on the FAU powder was considered to be consistent with the FAU-type zeolite membrane despite the differing Si/Al ratios. Figure 3 shows the TG-DTA measurements of the FAU powders after the immersion treatment of methanol, 1-hexanol, methyl hexanoate, and hexyl hexanoate. The FAU powder with non-immersed is presented as a reference. The non-immersed FAU powder showed a 23.5% mass loss from 300 K to 473 K with a corresponding endothermic DTA peak due to water desorption from the zeolite powder. Above 473 K, the mass and DTA curves were constant, suggesting the zeolite was stable.

The effect of methanol on the FAU powder is shown in Figure 3a. The TG curve of the FAU powder immersed in methanol shows two steps of mass loss at about 300–450 and 500–650 K. Similar to the reference, 21.0% mass loss was observed due to water desorption up to 450 K with a corresponding endothermic DTA curve. At higher temperatures, a smooth exothermic peak appeared around 530 K with an associated mass loss of 6.7%, which may be attributed to the combustion of methanol strongly adsorbed on the zeolite. Similarly, the FAU powder immersed in 1-hexanol, shown in Figure 3b, showed the first mass loss of 24.0% at 300–500 K due to water desorption and the second mass loss of 4.4% at 500–650 K due to the combustion of strongly adsorbed 1-hexanol, with an exothermic peak at 542 K.

The TG curves of the FAU powders immersed in methyl hexanoate and hexyl hexanoate showed three steps of mass loss (Figure 3c,d). The first mass losses for methyl hexanoate and hexyl hexanoate were 22.1% and 20.6% at 300–470 K, respectively, due to water desorption. For the methyl hexanoate immersed FAU powder, two exothermic peaks were identified at 470–650 K and 730–900 K with mass losses of 3.9% and 2.0%, respectively. These mass losses were attributed to the combustion of methyl hexanoate, with the difference in temperatures being attributed to different adsorption sites or partial decomposition of the methyl hexanoate molecule. Hexyl hexanoate showed the same trends as methyl hexanoate, as shown in Figure 3d. The second and the third mass losses of 8.1% at 470–770 K and 1.4% at 800–870 K were due to the decomposition of strongly adsorbed hexyl hexanoate, with corresponding exothermic DTA curves.

**Figure 4:**
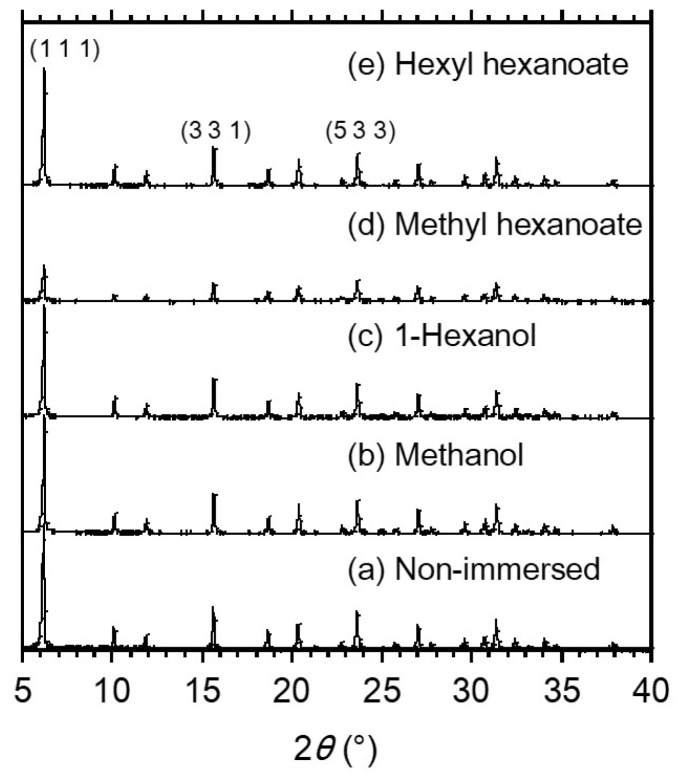
XRD pattens of the FAU-powder after immersion treatments in (**a**) non-immersed reference (**b**) methanol, (**c**) 1-hexanol, (**d**) methyl hexanoate, and (**e**) hexyl hexanoate.

XRD analysis was performed on the immersed FAU powders to check for structural changes to the zeolite by immersion treatments. Figure 4 shows the XRD patterns of the FAU powders after immersion treatment of methanol, 1-hexanol, methyl hexanoate, and hexyl hexanoate. Although all immersed FAU powders showed peaks at the same 2*θ*, the FAU powder immersed in methyl hexanoate had significantly decreased peak intensities. This suggests that the FAU-type zeolite structure was destroyed by methyl hexanoate.

Table 2 summarizes the XRD peak intensity ratios of FAU powder with and without immersion treatments to discuss the influence of methyl hexanoate on the crystal phases of FAU-type zeolite structure. Three large peaks at 2*θ* = 6.2, 15.6, and 23.6° were selected, which correspond to (1 1 1), (3 3 1), and (5 3 3) planes of FAU-type zeolite, respectively [16]. Except for the FAU powder immersed in methyl hexanoate, the peak intensity ratio of *I*_(1 1 1)/(5 3 3)_ and *I*_(3 3 1)/(5 3 3)_ were 3.1–3.5 and 1.1–1.2, respectively. In contrast, the FAU powder immersed in methyl hexanoate gave the intensity ratios of *I*_(1 1 1)/(5 3 3)_ and *I*_(3 3 1)/(5 3 3)_ were 1.6 and 0.8. This suggests that methyl hexanoate breaks the (1 1 1) and (3 3 1) planes faster than (5 3 3). According to the results of the TG-DTA analysis shown in Figure 3c,d, methyl hexanoate and hexyl hexanoate were strongly adsorbed on zeolite. However, the peak intensity ratios between the esters were a gap, and only methyl hexanoate damaged the zeolite structure. Iglesia et al. reported that LTA-type zeolite membrane after the esterification reaction of acetic acid and ethanol obtained a lower XRD intensity and sharpness [17]. They concluded the zeolite structure changed due to the instability of the zeolite to the reaction of acidic conditions. Here, the FAU-type zeolite membrane had high alumina content like the LTA-type zeolite membrane with the Si/Al ratio of 1, so the FAU-type membrane would be damaged by acid. Methyl hexanoate is hydrolyzed and produces water [18]. Methyl hexanoate has a water solubility of 1.33 g L^−1^ and is higher than that of hexyl hexanoate (9.5 × 10^−6^ g L^−1^). Hence, it could be that methyl hexanoate may have disrupted the zeolite structure by producing hexanoic acid via residual water in the zeolite.

### 3.3. Vapor Exposure Tests

**Figure 5:**
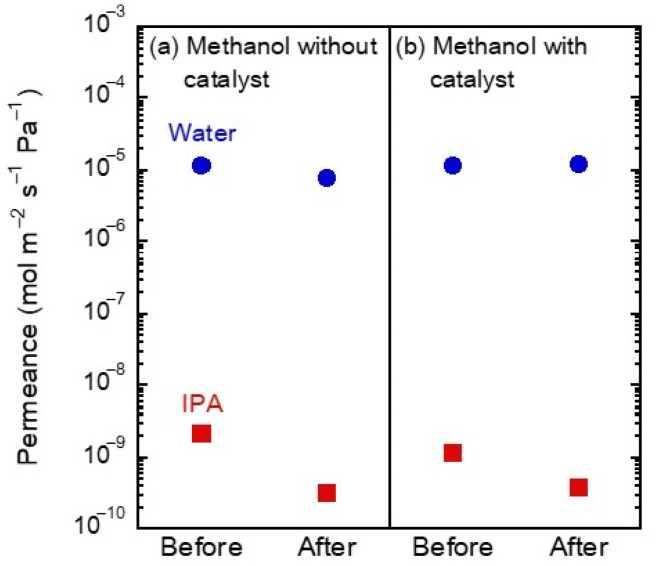
Water and 2-propanol permeances of the FAU-type zeolite membrane before and after methanol vapor exposure both (**a**) without catalyst and (**b**) with catalyst. Pervaporation performed with 10 wt% water/2-propanol at 348 K.

Firstly, the FAU-type zeolite membrane was exposed to methanol to check the influence of methanol on the permeation properties of the FAU-type zeolite membrane. Figure 5 shows the effect of methanol vapor exposure tests in the presence and absence of catalyst on water and 2-propanol permeance of the FAU-type zeolite membranes. The water permeance and separation factor of water/2-propanol were 1.0 × 10^−5^ mol m^−2^ s^−1^ Pa^−1^ and 6078 before the methanol vapor exposure test. After the exposure to methanol vapor without the catalyst, the water permeance and separation factor of water/2-propanol were 0.8 × 10^−5^ mol m^−2^ s^−1^ Pa^−1^ and 33,197, respectively. In the methanol vapor exposure test in presence of the catalyst, the changes in water permeance and separation factor before and after the test showed the same trends in the methanol vapor without the catalyst. Methanol is less adsorptive than esters from the discussion in Figure 3, and these results indicate that methanol is not adsorbed to the extent that it inhibits water permeation. Therefore, the FAU-type zeolite membrane was not affected by methanol vapor and the catalyst. Thereafter, the methanol permeation performance of FAU membranes before and after vapor exposure tests was evaluated by the methanol pervaporation test.

**Figure 6:**
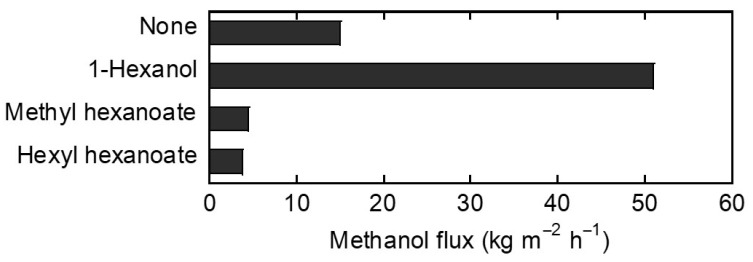
Methanol flux through FAU-type zeolite membranes at 333 K after 8 h vapor exposure to untreated, 1-hexanol, methyl hexanoate, and hexyl hexanoate.

Next, the influences of alcohols and esters were evaluated by the exposure to 1-hexanol, methyl hexanoate, and hexyl hexanoate vapor at 373 K for 8 h (Figure 6). The methanol flux of the untreated FAU-type zeolite membrane was 15.0 kg m^−2^ h^−1^. The methanol flux was increased to 50.9 kg m^−2^ h^−1^ by the exposure to 1-hexanol vapor. In contrast, the methanol fluxes were decreased by about 75% after exposure to methyl hexanoate or hexyl hexanoate vapors. Based on the discussion in Figure 3, these results suggest that methyl hexanoate and hexyl hexanoate adsorbed strongly on the FAU powder compared to methanol and 1-hexanol, which may explain why exposure to these esters can inhibit methanol permeation through FAU-type zeolite membranes.

Here, the molecular size against the pore size of FAU-type zeolite is discussed. The permeation fluxes from the quaternary vapor permeation tests for the mixture of methanol, 1-hexanol, methyl hexanoate, and hexyl hexanoate as the simulant transesterification reaction solution was evaluated, and hexyl hexanoate was not detected in the permeation stream [10]. The fluxes of 1-hexanol and methyl hexanoate were less than ten thousandth and thirty thousand of methanol flux, respectively, which suggests the order of the molecular size is 1-hexanol < methyl hexanoate < hexyl hexanoate. As shown in Figure 3c,d, the esters can adsorb strongly on zeolites. Therefore, esters are considered to adsorb on zeolite, which suggests that strong adsorption of esters may have inhibited the permeation of methanol. Besides, 1-hexanol was permeable through the FAU-type zeolite membrane since the adsorption of 1-hexanol was weaker than the esters discussed in Figure 3 and the molecular size of 1-hexanol (kinetic diameter: 0.62 nm [19]) was smaller than the pore of the FAU-type zeolite (0.74 nm [20]). Moreover, the increase of the methanol flux after 1-hexanol exposure was considered because of the removal of water adsorbed on the zeolite membrane by the vacuum at 373 K during the vapor exposure test. In the report of LTA-type zeolite membrane with Si/Al ratio = 1.5, the presence of water on the zeolite membrane inhibits methanol permeation through the zeolite membrane due to strong water adsorption in the zeolite pores [21]. As shown in Figure 5, the FAU-type zeolite membrane with Si/Al ratio = 1.3 showed high water/2-propanol separation factors, despite the size of 2-propanol (kinetic diameter: 0.47 nm [22]) being smaller than the FAU-type zeolite pore size. The high separation factor was likely due to adsorbed water preventing 2-propanol from permeating through the FAU-type zeolite membrane. These results indicate methanol permeation is inhibited by water adsorption on zeolite with high alumina content. Furthermore, after the FAU-type zeolite membrane was exposed to air in the feed with the permeate under vacuum at 373 K for 8 h, methanol flux was increased to 82.3 kg m^−2^ h^−1^, and yet the separation factor of water/2-propanol remained higher than 10,000 in the 10 wt% water/2-propanol pervaporation test. Thus, the removal of water from the zeolite by the vacuum at 373 K is considered to be the most likely reason for the increased methanol permeation through the FAU-type zeolite membrane. Based on these results, methanol flux increased after the vapor exposure test with 1-hexanol because 1-hexanol did not occlude the zeolite pores, and adsorbed water was removed during the test. Regarding the FAU-type zeolite membrane after methyl hexanoate exposure, the zeolite layer did not exfoliate from the support tube, suggesting the membrane structure was not destroyed.

**Figure 7:**
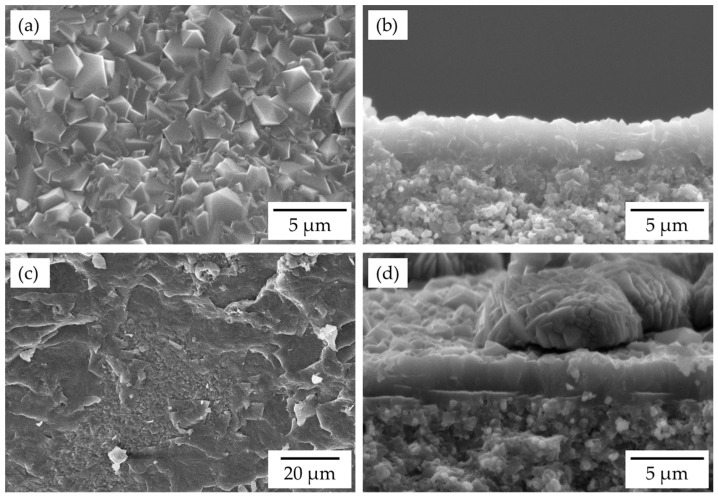
SEM images of the (**a**) surface and (**b**) cross-section of the non-exposed FAU-type zeolite membrane (5 SiO_2_:1Al_2_O_3_:17 Na_2_O:1000 H_2_O, 16 h synthesis), and the (**c**) surface and (**d**) cross-section of the FAU-type zeolite membrane after hexyl ether vapor exposure.

Additionally, the influences of hexyl methyl ether and hexyl ether on the methanol flux of the FAU-type zeolite membrane were evaluated. In the membrane-assisted transesterification reaction at 373 K, small amounts of ethers were produced by the side reactions of methanol and 1-hexanol. The FAU-type zeolite membranes for the exposure to the ethers were synthesized with a molar ratio of 5 SiO_2_:1 Al_2_O_3_:17 Na_2_O:1000 H_2_O at 363 K for 16 h. As shown in Figure 7a,b, the α-alumina support tube was completely covered with the polycrystalline layer having a grain size of 1–2.5 μm, and the thickness of the polycrystalline layer was 2.5–3.6 μm. The polycrystalline layer was obtained the Si/Al ratio of 1.35. The membrane properties were the same as the FAU-type zeolite membrane as shown in Figure 1.

**Figure 8:**
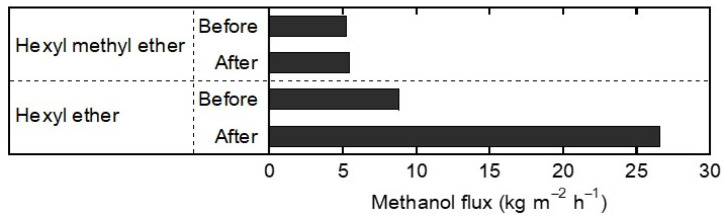
Effects of vapor exposure tests with hexyl methyl ether and hexyl ether on methanol flux of the FAU-type zeolite membranes (5 SiO_2_:1 Al_2_O_3_:17 Na_2_O:1000 H_2_O, 16 h synthesis).

Figure 8 shows the methanol fluxes at 333 K before and after exposure to the vapors of hexyl methyl ether and hexyl ether. The methanol flux after the exposure to hexyl methyl ether was almost identical to that before the exposure test. This indicates that hexyl methyl ether does not affect the methanol permeation of the FAU-type zeolite membrane. On the contrary, the methanol flux increased from 8.8 to 26.5 kg m^−2^ h^−1^ by exposure to hexyl ether.

The XRD patterns of the exposed FAU-type zeolite membranes were taken to check the influence on the zeolite structure by exposure to the ether vapors. Table 3 summarizes the XRD peak intensity ratios of the FAU-type zeolite membranes before and after exposure to hexyl methyl ether and hexyl ether. Four α-alumina peaks from the support tube at 2*θ* = 25.7, 35.3, 37.9, and 43.5° for comparison. The intensity ratios were calculated by dividing the intensities of (1 1 1), (3 3 1), and (5 3 3) planes by the sum of intensities of the four α-alumina peaks, respectively. Because the synthesis method was identical for each membrane, the membrane thickness was considered constant. Both of the exposed FAU-type zeolite membranes gave low-intensity ratios compared to the non-exposed FAU-type zeolite membrane. The peak intensity ratio of *I*_(1 1 1)_/*I*_α-alumina_ decreased from 0.27 for the unexposed membrane to 0.13 for hexyl methyl ether and 0.22 for hexyl ether. This suggests that hexyl methyl ether is more likely to break the zeolite structure, especially the (1 1 1) planes, than hexyl ether.

Since the FAU-type zeolite membrane exposure to hexyl ether vapor changed the membrane surface color from white to light yellow, it was observed by SEM (Figure 7c,d). At the center of the FAU-type zeolite membrane, as shown in Figure 7d, the α-alumina support tube was covered with a polycrystalline layer of FAU-type zeolite, with the same thickness of about 2.5 μm as before the vapor exposure test (Figure 7b). However, the surface scan of the membrane showed evidence of the uncovered support tube and the presence of the resin sealant, which was used to connect the membrane and stainless-steel tubing (Figure 7c). This implies that hexyl ether may have destroyed and washed away the zeolite layer and dissolved parts of the resin because hexyl ether is miscible in water and various organic solvents. So, operation below 373 K can prevent the ethers from attacking the zeolite or sealing resin due to no observation of side reactions.

Finally, the structural stability of the FAU-type zeolite membrane is discussed. Based on the XRD peak intensity ratios of the immersed FAU powder (Figure 4), only methyl hexanoate damaged the zeolite structure. When the FAU powder immersed in the mixture of methyl hexanoate and 1-hexanol (molar ratio = 1), the peak intensity ratios of *I*_(1 1 1)/(5 3 3)_ and *I*_(3 3 1)/(5 3 3)_ were 3.1 and 1.0, respectively. It suggests the FAU-type zeolite structure is not broken by reducing the concentration of methyl hexanoate and is maintained during the transesterification reaction. In addition to the destruction of the zeolite structure (Table 3), hexyl ether caused the zeolite layer to delaminate from the support tube. It suggests that FAU-type zeolite membranes would likely have long-term stability issues for reactions where ethers are produced. The authors’ previous work identified some membranes after transesterification that could be reused for 4 cycles and some that could not by the evaluation of 10 wt% water/2-propanol pervaporation after the reaction [10]. The most likely factor affecting reusability is concentration of ether produced at the operating temperature of 373 K.

## 4. Conclusions

The effects of alcohols and esters in the transesterification reaction on FAU-type zeolite membranes were examined by the immersion tests of the FAU powder in methanol, 1-hexanol, methyl hexanoate, and hexyl hexanoate. TG-DTA measurements with the immersed FAU powder suggest that methyl hexanoate and hexyl hexanoate strongly adsorb on the zeolite. Adsorption of methanol and 1-hexanol on the zeolite was weaker compared the esters, which suggests the esters may inhibit methanol permeation through FAU-type zeolite membranes due to preferential adsorption. Next, effects of methanol, 1-hexanol, methyl hexanoate and hexyl hexanoate on the permeance properties of FAU-type zeolite membranes were investigated. Methanol vapor exposure for 8 h at 373 K showed no influence on the water and 2-propanol separation performance. The organic vapor stability of the FAU-type zeolite membrane was evaluated by the methanol permeation of pervaporation at 333 K before and after the vapor exposure tests with 1-hexanol, methyl hexanoate, and hexyl hexanoate, respectively. Exposure to esters (methyl hexanoate and hexyl hexanoate) caused the methanol flux through the FAU-type zeolite membranes to decrease by a quarter. These results suggest that to develop an FAU-type zeolite membrane with high methanol flux during the reaction, the methanol permeance reduction due to exposure to esters will need to be inhibited.

## Figures and Tables

**Table 1:** Antoine constants and Wilson parameters of components of pervaporation test solutions [15].

Component	Antoine Constant			Wilson Parameter	
	A	B	C	Λ_AW_	Λ_WA_
Water	8.02754	1705.616	231.405	-	-
Methanol	8.07919	1581.34	239.65	0.55148	0.89781
2-Propanol	6.6604	813.055	132.93	0.04857	0.77714

**Table 2:** XRD peak intensity ratio of the FAU powders immersed in alcohols and esters.

Immersion Solution	Intensity Ratio (-)	
	*I*_(1 1 1)_/*I*_(5 3 3)_	*I*_(3 3 1)_/*I*_(5 3 3)_
None	3.3	1.1
Methanol	3.5	1.2
1-Hexanol	3.1	1.1
Methyl hexanoate	1.6	0.8
Hexyl hexanoate	3.4	1.1

**Table 3:** XRD peak intensity ratio of the FAU membranes immersed in the ethers.

Exposed Vapor	Intensity Ratio (-)		
	*I*_(1 1 1)_/*I*_α-alumina_	*I*_(3 3 1)_/*I*_α-alumina_	*I*_(5 3 3)_/*I*_α-alumina_
None	0.27	0.10	0.14
Hexyl methyl ether	0.13	0.07	0.09
Hexyl ether	0.22	0.09	0.12

## Data Availability

The data presented in this study are available on request from the corresponding author.
